# Carbapenem-Resistant *Klebsiella pneumoniae* Infections in ICU COVID-19 Patients—A Scoping Review

**DOI:** 10.3390/jcm10102067

**Published:** 2021-05-12

**Authors:** Wioletta Mędrzycka-Dąbrowska, Sandra Lange, Katarzyna Zorena, Sebastian Dąbrowski, Dorota Ozga, Lucyna Tomaszek

**Affiliations:** 1 Department of Anaesthesiology Nursing & Intensive Care, Faculty of Health Sciences, Medical University of Gdańsk, Dębinki 7, 80-211 Gdańsk, Poland; 2 Department of Anesthesiology and Intensive Care, Hospitals Tczewskie SA, 30 Stycznia 57, 83-110 Tczew, Poland; langa94@gumed.edu.pl; 3 Department of Immunobiology and Environment Microbiology, Faculty of Health Sciences, Medical University of Gdańsk, Dębinki 7, 80-211 Gdańsk, Poland; katarzyna.zorena@gumed.edu.pl; 4 Departament of Medical Rescue, Faculty of Health Sciences, Medical University of Gdańsk, Dębinki 7, 80-211 Gdańsk, Poland; sebastian.dabrowski@gumed.edu.pl; 5 Institute of Health Sciences, College of Medical Sciences of the University of Rzeszow, St. Warzywna1A, 35-310 Rzeszow, Poland; gdozga@poczta.fm; 6 Department of Specialist Nursing, Faculty of Medicine and Health Sciences, Kraków Academy of Andrzej Frycz Modrzewski, St. Gustawa Herlinga-Grudzińskiego 1, 30-705 Kraków, Poland; ltomaszek@igrabka.edu.pl

**Keywords:** carbapenem-resistance, *Klebsiella pneumoniae*, COVID-19

## Abstract

Introduction: The spread of multidrug-resistant pathogens is a serious problem and challenge for the whole medical community. Carbapenem-resistant *Klebsiella pneumoniae* (CRKP) infections in immunocompromised patients have a severe course and may be fatal. Increasingly, these bacteria are exhibiting resistance to carbapenem antibiotics, which have been used as so-called drugs of last resort. The emergence of the new coronavirus and the pandemic that it has caused require changes to protect against the spread of the new SARS-CoV-2. These changes paradoxically may contribute to the spread of other infections. Methods: PubMed, Cochrane Library databases were searched using relevant keywords. A literature review of carbapenem-resistant *Klebsiella pneumoniae* infection in patients hospitalized for COVID-19 was conducted according to PRISMA recommendations. A written review protocol was not prepared. Results: 1016 studies in scientific databases were searched. After rejecting duplicate studies, 964 results were obtained. Inclusion and exclusion criteria were then applied, and studies were qualitatively analyzed. Finally, 11 studies were included in the review. The results of infected patients were from six countries. The prevalence of CRKP in Covid-19 patients ranged from 0.35–53%. The majority of CRKP infected patients were male (85%), with a mean age of 61 years. Among isolates, the predominant genes were KPC, OXY-48, CTX-M, TEM, NDM and SHV. Conclusion: The results presented in our review indicate the necessity of paying attention to carbapenem-resistant *Klebsiella pneumoniae* infections in patients with COVID-19. In order to prevent the increase of bacterial resistance, rational antibiotic therapy should be used, as well as continuous control and surveillance of hospital infections caused by *multidrug-resistant organisms.*

## 1. Introduction

Bacterial resistance has become a worldwide problem. The spread of pathogens that are insensitive to multiple classes of antibiotics—multidrug-resistant bacteria (MDRB), especially in intensive care units (ICUs), is a serious problem and challenge for the whole medical community [1,2]. *Klebsiella pneumoniae* is a Gram-negative species included among Enterobacterales. The bacterium can reside in the gastrointestinal tract as a physiological component of the intestinal flora, on the skin, and in the oral cavity. Unfortunately, in immunocompromised patients, it can cause severe infections, including urinary tract infections, respiratory infections, soft tissue infections, peritonitis and sepsis [3,4,5,6]. The envelope, lipopolysaccharides (LPS) and cell wall protein receptors are responsible for the pathogenicity of *Klebsiella pneumoniae*. These factors determine the process of binding to host cells and provide protection against response from the human immune system [7,8]. Recently, these bacteria more and more frequently demonstrate resistance to antibiotics of the carbapenem group, which were used as so-called drugs of last resort (DoLR) in the course of severe infections caused by Gram-negative bacilli [6].

### 1.1. Resistance in Klebsiella pneumoniae

Resistance to carbapenem in Enterobacterales is created by two mechanisms. The first mechanism is represented by the production of enzymes that are able to hydrolyze cephalosporins (ESBL [extended-spectrum beta-lactamase] and AmpC beta-lactamases) [9,10] combined to functional alteration or loss of porins. A second, increasingly common mechanism is the production of β-lactamases capable of hydrolyzing most antibiotics, including carbapenems, called carbapenemases. These include: *Klebsiella pneumoniae* producing carbapenemases (KPC), metallo-beta-lactamases (NDM, IMP, VIM) and OXY-48-like carbapenemases [10,11]. KPC is a pathogen that has the capacity for clonal expansion and genetic exchange that contributes to resistance. Moreover, it can survive in human reservoirs and form biofilms that are resistant to disinfectants used in hospitals [12,13,14]. The mechanism of NDM-1 (New Delhi metallo-beta-lactamase-1) resistance was first described in 2009. The bacterium was isolated from urine. From a therapeutic standpoint, this is the most dangerous resistance mechanism, because the bacteria are resistant to almost all available antibiotics. The exceptions are colistin and tigecycline. In addition, the genes encoding NDM-1 show ease in transferring to other species of *Enterobacteriaceae* family strains [8,15].

### 1.2. Co-Infections in Patients with COVID-19

A new virus was defined by the International Committee on Taxonomy of Viruses (ICTV) as severe acute respiratory syndrome coronavirus (SARS-CoV-2). The disease was named COVID-19 (“coronavirus disease 2019”) by the World Health Organization (WHO). The number “19” in the abbreviation stands for the year 2019, when the virus was first observed [16]. The emergence of the new coronavirus and the pandemic that it caused required significant changes throughout the healthcare system [17,18]. The opening of temporary hospitals and intensive care units (ICUs), the necessity to work in personal protective equipment (PPE), work overload and isolation of health care workers (HCWs) necessitated the hiring of additional personnel, who were often not experienced in working in ICUs. In addition, the widespread use of broad-spectrum antibiotics, deficiencies in the availability of PPE equipment, and reduced infection control and prevention have led to the increasing emergence of multidrug-resistant bacteria [19,20]. Available data show that secondary infections in patients with COVID-19 occur in 4% to 15% of hospitalized patients, particularly in patients with severe COVID-19 infection, and are associated with increased mortality [21,22,23,24,25].

### 1.3. Objective

The aim of this study was to review the literature in available scientific databases on carbapenem-resistant *Klebsiella pneumoniae* (CRKP) bacterial infection in patients hospitalized for COVID-19 and to identify reasons that may contribute to the spread of MDROs (multi-drug resistant organisms) during the SARS-CoV-2 pandemic.

## 2. Methods

### 2.1. Study Design

Scoping review was conducted in the first quarter of 2021.

### 2.2. Definition of Scoping Review

Scoping reviews are a relatively new approach to synthesizing evidence, and there is currently little guidance on deciding between a systematic review and a scoping approach during the synthesis of evidence, especially when the literature has not yet been comprehensively reviewed or shows a large, complex or heterogeneous nature that cannot be subject to a more thorough systematic review [26].

### 2.3. Search Strategy

The following words were used to verify the search: carbapenem-resistance, *Klebsiella pneumoniae*, COVID-19. Keyword combinations with AND, OR and both operators were entered. The number of articles retrieved during each search test was limited to studies conducted between 2019 and 2021. Strict inclusion and exclusion criteria were applied (Table 1). The last search was conducted in March 2021. Finally, 10 articles were included in our review. The search was conducted by two experts in the field of scientific information in health sciences. Discrepancies were resolved through discussion.

### 2.4. Study Selection

Inclusion criteria: hospitalized patients for COVID-19 with a positive CRKP result. Exclusion criteria: non-COVID-19 patients, articles published in languages other than English and articles for which the full version could not be accessed.

### 2.5. Selection Process

The quality of articles selected for review was assessed using the Newcastle Ottawa Scale. Articles that were reviewed were given a score of 5–8 (Table 2). The AMSTAR 2 quality assessment checklist for systematic reviews and Preferred Reporting Items for Systematic Reviews and Meta-Analyses (PRISMA) [27,28,29] were used. This review does not include meta-analyses; any associated AMSTAR 2 or PRISMA checklist items were considered not applicable. A summary of the methodological quality assessment using the AMSTAR 2 checklist is presented in Table 3. The contents of two electronic databases, PubMed and the Cochrane Library, were searched.

### 2.6. Data Extraction

Studies were evaluated using a formalized form of data extraction that included the following data: first author, year of publication, country, study population, number of infected patients, resistance gene.

## 3. Results

### 3.1. Results of the Scoping Review

**Figure 1:**
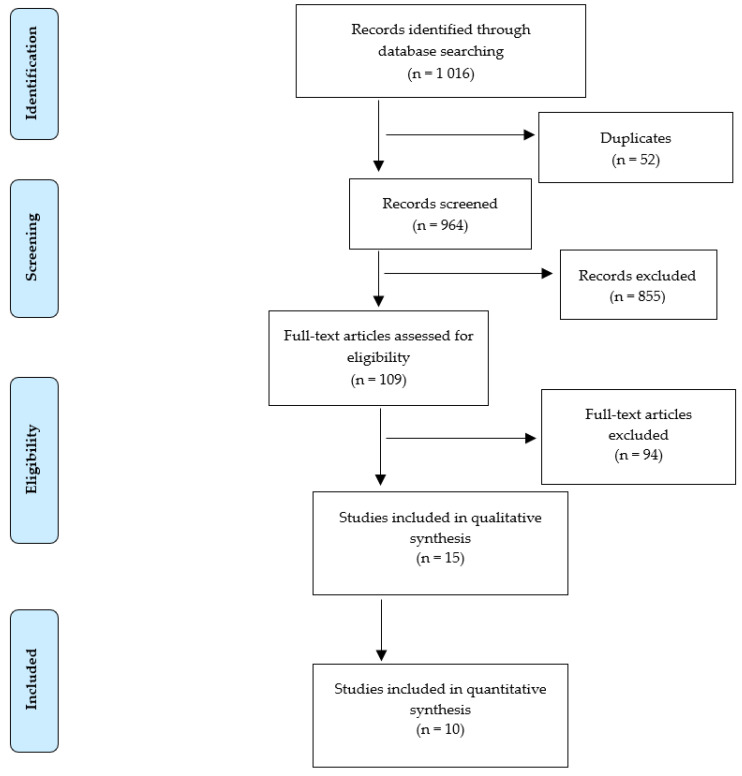
Flow diagram of study selection and inclusion.

Studies in which CRKP positivity was identified in patients hospitalized with COVID-19 were included in the review. A total of 1016 articles were found in scientific databases. After removing duplicates, 964 papers remained for analysis. In the next step, 109 full-text articles were retained after reviewing abstracts. The next step focused on inclusion and exclusion criteria (94 were rejected). At the stage of qualitative text analysis, four articles were rejected. Finally, 10 articles were accepted for systematic analysis (Figure 1).

### 3.2. Demographic and Social Data

Reports of the CRKP patients came from six countries. The cases were diagnosed in Italy (Napoli, Umbria, Turin, Rome, Genoa), China (Wuhan), Egypt (Assiut), United States (New York City), Spain (Oviedo, Asturias), and Peru. The prevalence of infected CRKP in Covid-19 patients ranged from 0.35% to 53%. The distribution of cases for which sex was available was as follows: 5 women (16%) and 26 men (84%). For patients with reported age, the mean age was 61 years, ranging from 23 to 76 years. These data are presented in Table 4 and Table 5.

### 3.3. Characteristics of the Study Population

Sixteen (50%) patients in the study by Karruli A. et al. developed MDR infection in the ICU. Patients who were found to be colonized with MDRB were not included in the group of patients with MDR infection. A total of 23 isolates were cultured, of which the most common pathogens were carbapenem-resistant *K. pneumoniae* (32%) and *A. baumannii* (19%) Eight *K. pneumoniae* isolates were resistant to carbapenems, all due to KPC-type carbapenemase production. The most common infectious syndromes caused by MDR pathogens were bloodstream infection and ventilator-associated pneumonia [30]. In a single-centered, retrospective, observational study by Yang X. et al., hospital-acquired infection was reported in 7 of 52 hospitalized patients. Moreover, one patient (2%) had pulmonary infection and blood stream infection of CRKP [31]. Li J. et al., in their study on the etiology and resistance of secondary bacterial infections in COVID-19 patients, sampled cultures from 102 patients. Furthermore, 35 (34.3%) patients had *K. pneumoniae*, of which 32 (31.4%) were resistant to carbapenems. The main type of infection was pulmonary, followed by bloodstream infections [32]. As for Ramadan, in H.K.A. et al.’s study, in 28 cases, bacterial and/or fungal co-infections were found in respiratory samples. The total number of clinical isolates obtained was 42, of which 37 were bacteria. Furthermore, 12 of these were *K. pneumoniae* [33]. Gomez-Simmonds, A. et al., among 3152 patients with COVID-19, identified 13 patients positive for CRE, including 11 with *K. pneumoniae*—KPC. The main source of infection was the respiratory tract. Additionally, 7/13 CRE cases (54%) subsequently developed bacteremia [34]. In a study presented by García -Menioño I. et al., 62 patients were treated in the ICU for COVID-19, and none of these patients were colonized before admission. From clinical and epidemiological surveillance samples, 7 (11.3%) patients positive for CRKP were identified; 4 were colonized, 2 developed VAP and 1 patient developed primary bacteremia [35]. Among 35 patients, 7 (20%) had a positive rectal swab for *carbapenemase-producing K. pneumoniae* in a Montrucchioa, G. et al. study. One patient became colonized in the unit and 6 developed invasive infection [36]. Overall, 65/80 patients hospitalized in the ICU for COVID-19, in a study by Arcari, G. et al., were screened with smears for *carbapenemase-producing Enterobacterales* colonization. Positive cultures for *carbapenemase-producing K. pneumoniae* were found in 14 patients (22%). Seven patients developed co-infection that was confirmed in 5 bronchoalveolar lavages and 2 blood cultures [37]. In a Magnasco, L. et al. study, 2 ICU patients were colonized with CRKP. In one, colonization occurred after transplantation and subsequently developed into ventilator-associated pneumonia (VAP). The other one also developed VAP caused by both CRKP and CRPA (carbapenem-resistant *P. aeruginosa*] [38]. Arteaga-Livias, K. et al. described 4 cases of MDR *K. pneumoniae*; two of these developed co-infection [39].

## 4. Discussion

The review data on identification of Carbapenem-resistant *K. pneumoniae* are from different continents, i.e., Europe, Asia, North and South America and Africa. The prevalence of coinfection in COVID-19 patients ranged from 0.35% to 53%. The majority of CRKP patients were males with a mean age of 61 years. The most frequently isolated resistance gene was KPC, followed by OXY-48, CTX-M, TEM, NDM, and SHV. The main type of infection was pulmonary and bloodstream. This may be associated with the use of mechanical ventilation and central catheters in ICUs. Li J. et al., in their study, showed that the incidence of invasive mechanical ventilation and central catheter placement was higher in the critically ill group. At the same time, a higher incidence of secondary bacterial infections was observed in this group [32]. Reducing the spread of MDROs in medical facilities and particularly in ICUs has become a challenge for the medical community. In many countries, infection control programs have been implemented to prevent the colonization and infection of patients. In accordance with guidelines of the European Society of Clinical Microbiology and Infectious Diseases (ESCMID), educational training on hand hygiene, patient contact precautions, the implementation of a rapid pathway to identify and isolate patients colonized or infected with the bacterium, and a screening culture procedure have been initiated [6,14,31]. The emergence of the new SARS-CoV-2 coronavirus in 2019 has necessitated additional precautions. In order to minimize the spread and infection with the virus, all ward staff were equipped with PPE such as coveralls, goggles, masks, leggings, and several pairs of gloves. Although the steps taken were supposed to reduce the risk of spreading the new virus, paradoxically, they may have favored the spread of other multidrug-resistant bacteria [35,39]. In a study by Tiri et al. (Umbria, Italy), CRE (carbapenem-resistant Enterobacteriaceae) cases increased from an average of 6.7% to as much as 50%, during the pandemic, when the ICU was designated exclusively for intubated COVID-19 patients. It is important to highlight the fact that the use of PPE protected staff from contracting the virus. None of the workers became SARS-CoV-2 positive [40]. A study by Belvisi V. et al. also showed an increasing trend in the prevalence of the fecal carriage of *K*. *pneumoniae*—KPC during the pandemic. After implementation of anti-KPC program, the prevalence of *K. pneumoniae*—KPC colonization in the ICU decreased from 71.4 to 0% and in the contiguous sub-intensive EM (emergency medicine) from 42.9% to 11.1%. In March, the hospital was assigned to hospitalize COVID-19 patients and the training program against *K. pneumoniae*—KPC mainly focused on PPE. Consequently, an increase in the prevalence of *K. pneumoaniae*—KPC carriage was recorded in the following months [41]. Chinese researchers—Li J. et al. showed in their study that *A. baumannii*, *K. pneumoniae*, and *S. maltophilia* were the main causes of secondary bacterial lung infections in COVID-19 patients, where the etiology is significantly different from the pre-pandemic of COVID-19 [32]. García-Menioño et al. (Spain), who identified the occurrence of the OXA-48 gene among their patients, highlighted the fact that, so far, none of the patients had been previously colonized by Enterobacteriaceae producing this gene [35]. Furthermore, Arcari et al. (Italy) pointed out that while the occurrence of KPC strains has been described in their country, the OXY-48 gene has rarely been reported [37]. Ramadan et al., who in their study undertook the characterization of patients with COVID-19 from Upper Egypt, noted that no co-infections were observed in patients in the mild severity group. They occurred only in the group with moderate and severe courses of COVID-19. Additionally, these cases were associated with greater severity and respiratory complications [33]. Similarly, in the study by Gomez-Simmonds et al., 12/13 patients with positive CPE bacteria (11- Kp-KPC, 2 NDM-E. *cloacae complex*) required intubation and intensive care [34]. In 5/7 patients presented by Montrucchio et al., septic shock occurred [38]. Similarly, in a study by Li J. et al. secondary bacterial infection was more likely to develop in the critical versus severe patient group, 26.7% (69/258) vs. 3.1% (33/1050). Furthermore, 49% of patients who acquired a bacterial infection died during hospitalization. The mortality rate of patients with acquired infection was also significantly higher in the critically ill group, 65.2% (45/69) vs. 15.2% (5/33) [31]. In a study by Soriano MC., the ICU mortality rate in the group of patients with ward-acquired infection was higher than in the group of patients without infection. They were 75.0% (15/20) and 44.4% (28/63), respectively [42]. However, researchers Karruli A. et al. pointed out that MDR infection was a common complication in ICU COVID-19 patients. However, their study showed that it appeared as a late complication associated with longer ICU stays [30]. The steadily increasing number of SARS-CoV-2 infections has necessitated the opening of temporary hospitals and ICUs dedicated only to COVID-19 patients. Several authors have hypothesized that the contaminated PPE of medical workers may be the main cause of cross-transmission. Factors such as using the same protective facemasks to care for different patients on the same unit and the use of double gloves where the outer gloves were changed and the inner gloves were disinfected with alcohol may have been causative factors. Although the unit implemented a procedure to use additional layer of fabric or plastic disposable gowns, this did not prevent the occurrence of further MDR infections [31,35,39]. Another reason for the spread of CRKP may have been the need to employ additional medical staff who often had no experience of working in the ICU (trainee doctors, doctors and nurses from other departments, physiotherapists, volunteers) [31,39]. Tiri et al. observed an interesting phenomenon in patients where positioning (prone position) was used in the treatment process. In their study, 67% of patients who were repositioned developed CRE colonization, whereas among patients who were not prone-positioned, the percentage was 37% [31]. An important aspect of bacterial dissemination is the widespread use of broad-spectrum antibiotics in COVID-19 patients [34,39]. Given the small number of scientific studies on the occurrence of co-infections in hospitalized COVID-19 patients, it is challenging to differentiate between a viral infection due to COVID-19 and the presence of a bacterial infection based on clinical data [43,44]. Although antibiotic therapy for COVID-19 infection is not effective, antibiotics are prescribed to many patients. There are several studies that show that approximately 70% of patients hospitalized for COVID-19 received broad spectrum antibiotics [19,45,46]. Chedid M. et al., in their review of antibiotic therapy in COVID-19 patients, showed that the frequency of antibiotic use was 74%. They noted a trend toward antibiotic use in patients with mild to moderate disease. Of the patients who received antibiotics, only 17.6% had a secondary infection. Lansbury et al. in their review found co-infection in 14% of patients admitted to the wards and in 7% of hospitalized patients [47]. Rawson TM. et al. showed that 72% of patients received broad spectrum antibiotics. Only 8% of COVID-19 patients had bacterial or fungal co-infection identified [17]. These findings may raise concerns of overuse of antibiotic therapy and a subsequent contribution to the development of increased bacterial resistance [19,43]. Therefore, further research on co-infections is needed to provide rational antibiotic therapy in the era of the SARS-CoV-2 pandemic [48,49]. It is also important to remember that altered, difficult working conditions and fatigue can cause burnout among HCWs, thereby decreasing commitment to their work and adherence to infection prevention and control. Additionally, the PPE use may give an apparent sense of security to medical workers and contribute to the negligence of infection control [31,35,39]. The authors agree that infection control measures should be increased to minimize the spread of CRE. Loosening the rules of prophylaxis and surveillance of infections, irrational use of antibiotics may not only negatively affect the treatment process and mortality of patients with COVID-19, but also lead to the outbreak of a pandemic of bacterial infections caused by CRE. Not without reason, the authors give alarming titles to their publications: “…The storm after the storm, “…Keep an eye on the ball”, “…One Step Back in Antimicrobial Stewardship”, “…What Did Not Work?” [31,35,36,38].

## 5. A Limitation of Scoping Review

This review is a first step in expanding knowledge in this area. Further studies should be conducted using more extensive data and more detailed designs. The decision to include studies that identified only CRKP does not provide a view on the overall rate of CRE in patients hospitalized for COVID-19. In some presented studies, the discrimination between colonization and infection was not clear enough [31,34,38]. Although the use of broad spectrum antibiotics is the major drive for MDR pathogens, four (out of ten), studies did not mention their use in COVID 19 patients prior to MDR organism infection [31,33,37,38]. Studies from Italy are overpresented. It may be due to the fact that, according to the European Center for Disease Control, 7.5% of carbapenem-resistant *K. pneumoniae* were isolated from cultures in Europe before the pandemic, while in Italy, it was 26.8%. At that time, infection control and prophylaxis programs were put to the test. In the course of the pandemic in Italy, control of bacterial infections took a back seat and they saw an increase in infections again. It should also be noted that the nature of the relatively new SARS-CoV-2 virus is still under investigation and data on it are constantly being updated.

## 6. Conclusions

The results presented here indicate the need for attention to infections caused by carbapenem-resistant *Klebsiella pneumoniae*. It is particularly true in patients with COVID-19, in whom the immune mechanisms seem to be weakened by this viral infection. Rational antibiotic therapy should be pursued with the goal of not allowing bacterial resistance to increase. There is also a need for the ongoing surveillance and control of hospital-acquired infections. These should not only focus on minimizing the spread of SARS-CoV-2 infection, but also on reducing bacterial cross-transmission, particularly of MDR organisms. The critically ill patients are the most susceptible to infection. Therefore, it should not be forgotten to continue the implementation of prophylaxis against ventilator-associated pneumonia, as well as bloodstream infections related to the poor management of central catheters.

## 7. Implications for Practice

Further research should be conducted on the causes of cross-transmission of infective organisms, with particular attention paid to the contaminated PPE of medical workers and patient positioning (prone position), as well as their impact on CRE colonization.

## Figures and Tables

**Table 1:** Inclusion and exclusion criteria, search strategies.

	Inclusion Criteria	Exclusion Criteria
**Years considered/Time period**	All evidence published in the last 2 years, period 2019–2021	
**Setting**	Healthcare settings for COVID-19 patients	Other healthcare settings
**Type of study design/references**	Observational studies, cases report, letters to the editor	Descriptive studies, single-case report
**Participants**	Positive COVID-19 patients	Negative COVID-19 patients
**Interventions**	Interventions to detect CRKP infection	Studies without a clearly described intervention
**Outcome measures**	Patient outcomes, culture outcomes	General outcome of co-infections in COVID-19 patients (without specification)
**Language**	English	Other lenguage
**Databases**	MEDLINE (PubMed), Cochrane Library	Other databases
**Key words**	Carbapenem-resistance, *Klebsiella pneumoniae*, COVID-19	
**Additional search terms, with which the central search terms were combined**	COVID-19, co-infections, Intensive Care Units *Klebsiella pneumoniae*, Carbapenem-Resistant Enterobacteriaceae, COVID-19 COVID-19, CRKP, ICU	

**Table 2:** Quality assessment of the included studies by the Newcastle–Ottawa Scale.

First Author, Year	Study Design	Selection	Comparability	Outcome	Total Scores
Karruli A. et al. 2019 [30]	Retrospective study	**	**	**	6
Yang X. et al. 2020 [31]	Retrospective study	***	**	*	6
Li J. et al. 2020 [32]	Retrospective study	***	*	**	6
Ramadan, H.K.A et al. 2020 [33]	Prospective study	***	**	**	7
Gomez-Simmonds, A. et al. 2020 [34]	Retrospective study	****	**	***	8
García–Menioño, I. et al. 2021 [35]	Retrospective study	***	**	***	8
Montrucchioa, G. et al. 2020 [36]	Retrospective study	***	**	***	8
Arcari, G. et al. 2021 [37]	Retrospective study	**	**	***	7
Magnasco, L. et al. 2021 [38]	Retrospective study	***	*	***	7
Arteaga-Livias, K. et al. 2021 [39]	Prospective study	***	**	**	7

* A study can be awarded a maximum of one star for each numbered item within the Selection and outcome categories (categories Selection max. 4 stars; categories Comparability max. 2 stars; categories Exposure/Outcome max. 3 stars).

**Table 3:** Summary of the AMSTAR 2 quality assessment.

First Author, Year	(1) Question and Inclusion	(2) Protocol	(3) Study Design	(4) Comprehensive Search	(5) Study Selection	(6) Data Extraction	(7) Excluded Studies Justification	(8) Included Studies Details	(9) Risk of Bias (RoB)	(10) Funding Sources	(11) Statistical Methods	(12) RoB on Meta-Analysis	(13) RoB in Individual Studies	(14) Explanation for Heterogeneity	(15) Publication Bias	(16) Conflict of Interest
Karruli A. et al. 2019 [30]	Yes	No	Yes	Yes	Yes	Yes	Yes	Yes	Yes	No	Yes	No	Yes	Yes	Yes	No
Yang X. et al. 2020 [31]	Yes	No	Yes	Yes	Yes	Yes	Yes	Yes	Yes	No	Yes	No	Yes	Yes	Yes	No
Li J. et al. 2020 [32]	Yes	No	Yes	Yes	Yes	Yes	Yes	Yes	Yes	No	Yes	No	Yes	Yes	Yes	No
Ramadan, H.K.A. et al. 2020 [33]	Yes	No	Yes	Yes	Yes	Yes	Yes	Yes	Yes	No	Yes	Yes	Yes	Yes	Yes	No
Gomez-Simmonds, A. et al. 2020 [34]	Yes	No	Yes	Yes	Yes	Yes	Yes	Yes	Yes	Yes	N/a	N/a	Yes	Yes	No	No
García—Menioño, I., et al. 2020 [35]	Yes	No	Yes	Yes	Yes	Yes	Yes	Yes	Yes	Yes	N/a	N/a	Yes	Yes	No	No
Montrucchioa, G. et al. 2020 [36]	Yes	No	Yes	Yes	Yes	Yes	Yes	Yes	Yes	No	N/a	N/a	Yes	Yes	No	No
Arcari, G., et al. 2021 [37]	Yes	No	Yes	Yes	Yes	Yes	Yes	Yes	Yes	Yes	N/a	N/a	Yes	Yes	No	No
Magnasco, L. et al. 2021 [38]	Yes	No	Yes	Yes	Yes	Yes	Yes	Yes	Yes	No	Yes	No	Yes	Yes	Yes	No
Arteaga-Livias, K. et al. 2021 [39]	Yes	No	Yes	Yes	Yes	Yes	Yes	Yes	Yes	No	N/a	N/a	Yes	Yes	No	No
**Total, N (%)**	**10 (100%)**	**0 (0%)**	**10 (100%)**	**10 (100%)**	**10 (100%)**	**10 (100%)**	**10 (100%)**	**10 (100%)**	**10 (100%)**	**3 (27%)**	**5 (50%)**	**1 (9%)**	**10 (100%)**	**10 (100%)**	**5 (45%)**	**0 (0%)**

**Abbreviations:** N/a—not applicable, RoB, Risk of Bias. Percent is based on number of eligible reviews per domain.

**Table 4:** Demographic and social data.

First Author, Year	Sex (%)	Age * [Years]
Karruli A. et al. 2019 [30]	n/d	n/d
Yang X. et al. 2020 [31]	n/d	n/d
Li J. et al. 2020 [32]	n/d	n/d
Ramadan, H.K.A. et al. 2020 [33]	n/d	n/d
Gomez-Simmonds, A. et al. 2020 [34]	1 F (9) 10 M (91)	62 (23–74)
García–Menioño, I. et al. 2020 [35]	1 F (14) 6 M (86)	67 (54–76)
Montrucchioa, G. et al. 2020 [36]	3 F (43) 4 M (57)	57 (41–71)
Arcari, G. et al. 2021 [37]	n/d	n/d
Magnasco, L. et al. 2021 [38]	0 F (0) 2 M (100)	65 (63,66)
Arteaga-Livias, K. et al. 2021 [39]	0 F (0) 4 M (100)	56 (45–66)

* average, F–Female, M–Male, n/d–no data.

**Table 5:** Analysis of articles included in the scoping review.

First Author, Year	Country (Region)	Population	Number of Infected Patients (%)	Resistance Gene
Karruli A. et al. 2019 [30]	Italy (Napoli)	32	n/d	KPC
Yang X. et al. 2020 [31]	China (Wuhan)	52	1(2%)	n/d
Li J. et al. 2020 [32]	China (Wuhan)	102	32 (31.4%)	n/d
Ramadan, H.K.A. et al. 2020 [33]	Egypt (Assiut)	260	n/d	KPC, CTX-M, TEM, SHV
Gomez-Simmonds, A. et al. 2020 [34]	United States (New York City)	3152	11(0.35%)	KPC
García–Menioño, I. et al. 2020 [35]	Spain (Oviedo, Asturias)	62	3(4.8%)	OXA-48, CTX-M
Montrucchioa, G. et al. 2020 [36]	Italy (Turyn)	35	6(17.1%)	KPC
Arcari, G. et al. 2021 [37]	Italy (Rome)	65	7(10.8%)	KPC, OXY-48
Magnasco, L. et al. 2021 [38]	Italy (Genoa)	118	2(1.7%)	n/d
Arteaga-Livias, K. et al. 2021 [39]	Peru	n/d	4	NDM, CTX-M

n/d—no data.

## Data Availability

The authors declare that the data of this research are available from the correspondence author on request.
